# Comparison between Areas of Bone Visualization Using Radiolucent Hybrid Fixator Frames and Graphically Simulated Metallic Frames: An Ex Vivo Study

**DOI:** 10.3390/vetsci9030120

**Published:** 2022-03-07

**Authors:** Andrea Bonardi, Gian Luca Rovesti, Filippo Maria Martini, Francesco Dondi, Davide Benedini, Fabio Barbieri

**Affiliations:** 1 Ortovet stp srl, Piazza Alessandrini 2/D, 43036 Fidenza, Italy; andrea.bonardi@ortovet.org; 2 Clinica Veterinaria M. E. Miller, Via della Costituzione 10, 42025 Cavriago, Italy; gl.rovesti@clinicamiller.it; 3 Department of Veterinary Science, University of Parma, Via del Taglio 10, 43126 Parma, Italy; filippomaria.martini@unipr.it; 4 Department of Veterinary Medical Sciences, Alma Mater Studiorum, University of Bologna, Via Tolara di Sopra 50, 40064 Ozzano dell’Emilia, Italy; f.dondi@unibo.it; 5 Ambulatorio Veterinario Dr. Lelio Benedini, Viale dell’Industria 97, 36071 Arzignano, Italy; 6 Freelance, Via G. Varesi 19, 43100 Parma, Italy; fabio.dr.barbieri@gmail.com

**Keywords:** hybrid external fixator, bone visualization, radiolucent materials, radiographic interference

## Abstract

The objective of this study is to evaluate the difference between the amount of bone visible with the superimposition of a radiolucent hybrid external fixator and a graphically simulated metallic frame. Eighteen frames were applied to eighteen bone specimens. The fracture area (FA), the radiolucent area (RLA) and the radiopaque area (ROA) inside the FA were calculated for each construct on both postoperative views. The ratio between the RLA and FA and between the ROA and FA was used to evaluate the amount of bone visible in the FA with a radiolucent and a radiopaque fixator, respectively. Finally, the areas of RLA and ROA were compared using the Wilcoxon test and Friedman test to evaluate the effect of the radiolucent material on the amount of bone visible. Differences were considered significant if *p* < 0.5. In every specimen *p* was <0.5. The amount of bone visible was significantly higher with the radiolucent frame compared to the radiopaque frame. Based on the results of this study, the use of radiolucent materials can be a valuable option for external fixation, in order to decrease the radiographic interference of the frame, allowing better assessment of fracture reduction and bone healing on postoperative radiographs.

## 1. Introduction

The circular (CEF) and hybrid (HEF) external skeletal fixation have been shown to be effective treatment modalities for fracture stabilization, for performing bone transport, limb lengthening and for the correction of angular and rotational limb deformities [1,2].

CEF and HEF both require limited surgical exposure and minimize disruption of the blood supply to bone and soft tissues [1,3]. They are useful for stabilizing highly comminuted fractures that cannot be anatomically reconstructed, while facilitating the management of associated soft tissue injuries. HEF constructs use the advantageous aspects of both linear external fixation (LEF) and CEF systems. The inherent shear stiffness of the hybrid constructs is attributed to the use of half-pins, while axial micromotion, typical of circular fixation, is preserved by the circular construct [4,5]. The circular component allows small peri- and juxta-articular fracture fragments to be transfixed and stabilized with small-diameter tensioned K-wires, while standard half-pins are used in the linear component of the frame [6,7]. The disadvantages in using an external fixator for fracture repair include the need for more postoperative (PO) care, the weight and size of the frame and the potential risk of infection along the pin and wire tracts [8]. One of the most important limitations associated with the use of external fixation is the radiographic interference due to the superimposition of the frame on the bone segment. This is particularly true with the use of CEFs that completely surround the limb. This can cause a difficult PO evaluation of fracture healing, requiring multiple radiographic projections, often oblique, which increase the exposure for both the patient and the personnel with poor repeatability that makes the comparison between different projections difficult [9,10].

Radiolucent materials for external fixation have been proposed in human medicine in order to overcome this problem. Furthermore, radiolucent (RL) fixators allow an easier reduction of the fracture under fluoroscopic-assisted intraoperative procedures [11,12,13,14,15].

The aim of this study was to compare the radiographic interference of a partially radiolucent external hybrid fixator applied to a cadaveric bone specimen with the same frame structure, graphically changed as if it was metallic. There is no reference to any specific fracture configuration; instead, the fracture was considered as potentially involving the whole area where the study is focused on. Since the bone specimens are not fractured, the study does not evaluate the correctness of the fixator design for the treatment of specific fractures, because the evaluation of technical aspects of fracture stabilization is beyond the scope of this work, and was already done in previous studies [5,10].

The null hypothesis was that there is no difference between the radiolucent and the software-generated, metallic-like frame in the amount of bone visible in the radiographs of the apparatus tested.

## 2. Materials and Methods

The bones used for the study were harvested from three dogs (*n* = 3) weighing between 24 and 36 kg that had died for reasons unrelated to this study. Bone segments were radiographed to exclude the presence of any orthopedic condition that would have interfered with subsequent evaluations. Soft tissues were removed and a total of 18 bones were obtained: six radius-ulna, six humeri and six tibiae. For each bone segment, two orthogonal preoperative radiographic projections, in DICOM format, were obtained in combination with a radiopaque marker of known size. The area of interest for radiographic evaluation is the fracture area (FA) that represent for each bone the area where the fracture is supposed to be. The FA was determined to define the area that would have been examined in order to measure the extent of radiographic interference by the frame. For each bone segment, the extent of the FA was determined in accordance with the following scheme.

**Figure 1:**
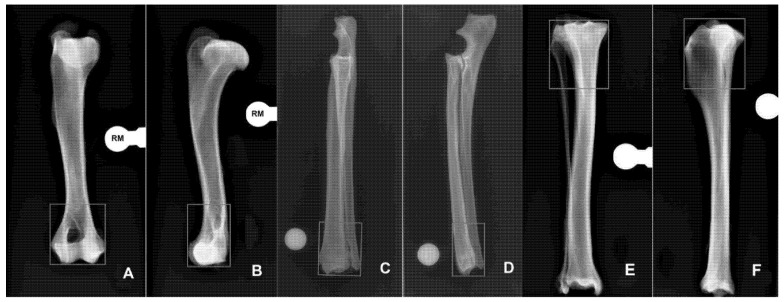
The fracture area (FA) of the humerus is represented by the distal quarter of the bone in both the CrCd (**A**) and ML (**B**) projections. The distal boundary is a line tangent to the joint, while the proximal one is a quarter of the way along the humeral length. The two connecting lines are parallel to the long axis of the humerus and tangent to the bone. The fracture area (FA) of the radius-ulna is represented by the distal quarter of the radial length in both the CrCd (**C**) and ML (**D**) projections. The distal boundary is a line tangent to the radial styloid, while the proximal one is a quarter of the way along the radial length. The two connecting lines are parallel to the long axis of the radius and tangent to the bone. The fracture area (FA) of the tibia is represented by the proximal quarter of the bone length in both the CrCd (**E**) and ML (**F**) projections. The proximal boundary is a line tangent to the joint, while the distal one is a quarter of the way along the tibial length down to the medial malleolus. The two connecting lines are parallel to the long axis of the tibia and tangent to the bone. In each bone segment, the FA represents the area where the fracture is supposed to be. Note that the radio-opaque marker (RM) is visible in each projection.

Humerus: the FA includes the humeral condyle and the supracondylar area considering the distal quarter of the bone length, measured from the greater tubercle to the joint line of the humeral condyle (Figure 1A,B).

Radius-ulna: the FA includes the distal quarter of the length of the radius, measured from the radial head to the styloid process (Figure 1C,D).

Tibia: the FA includes the proximal quarter of the tibia, calculated from the proximal edge of intercondylar eminences to the medial malleolus (Figure 1E,F).

The hybrid external radiolucent fixator was applied to each bone segment with the frame layout for the area that was supposed to be involved, following the frame layouts proposed in a previous study [16], in accordance with the following scheme.

**Figure 2:**
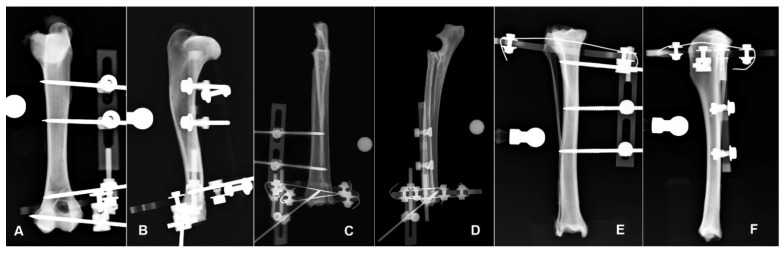
Hybrid frame application to the humerus for potential stabilization of a fracture in the planned fracture area (FA) in the CrCd (**A**) and ML (**B**) radiographic view. Hybrid frame application to the radius-ulna for potential stabilization of a fracture in the planned fracture area (FA) in the CrCd (**C**) and ML (**D**) radiographic view. Hybrid frame application to the tibia for potential stabilization of a fracture in the planned fracture area (FA) in the CrCd (**E**) and ML (**F**) radiographic view. Note that in each bone segment, the bone is still visible underneath the frame structure in both projections.

Humerus: The frame applied was made by a 180° carbon fiber ring of 84 mm inner diameter and 6 mm thick (Ad Maiora, Cavriago, Italy) The opening of the partial ring was oriented medially, the ring was orthogonal to the long axis of the bone and was connected to a 3 mm-threaded pin, which engaged the humeral condyle. A second threaded pin was connected to the ring and inserted just proximal to the supratrochlear foramen. A double slot radiolucent plastic post was connected to the upper side of the partial ring and supported two 3 mm-threaded pins inserted into the humeral diaphysis. Finally, the most proximal positive threaded pin engaging the diaphysis and the supratrochlear pin were connected by a linear carbon bar 5 mm in diameter and 250 mm long (Figure 2A,B) [16,17].

Radius-ulna: The hybrid frame applied to the radius was made by a 360° carbon ring of 84 mm inner diameter and 6 mm thick (Ad Maiora company, Cavriago, Italy), stabilized to the bone by two K-wires 1.5 mm in diameter, angled at about 60° and tensioned at 500 N, just proximal to the radio-carpal joint. The ring was orthogonal to the long axis of the bone. A single slot plastic post (Ad Maiora, Cavriago, Italy) was connected to the lower side of the ring and supported a 3 mm-threaded pin that engaged the distal epiphysis in the cranio-medial to caudo-lateral direction. Another plastic double post was connected to the upper side of the ring and supported two 3 mm-threaded pins inserted into the radial diaphysis (Figure 2D,E) [16].

Tibia: The hybrid frame applied to the tibia was made by a 270° carbon ring of 84 mm inner diameter and 6 mm thick (Ad Maiora, Cavriago, Italy). The partial ring was oriented with the opening caudally and was connected to the tibia with a 1.5-mm K-wire at the level of the proximal metaphysis oriented in the cranio-lateral to caudo-medial direction. From the upper side of the partial ring a 3 mm-threaded pin engaged the proximal metaphysis from the cranio-medial to the caudo-lateral aspect of the bone. The frame extended distally on the medial side with a double slot plastic post that supported two 3 mm-threaded pins engaging the tibial diaphysis in the medio-lateral direction (Figure 2E,F) [16].

**Figure 3:**
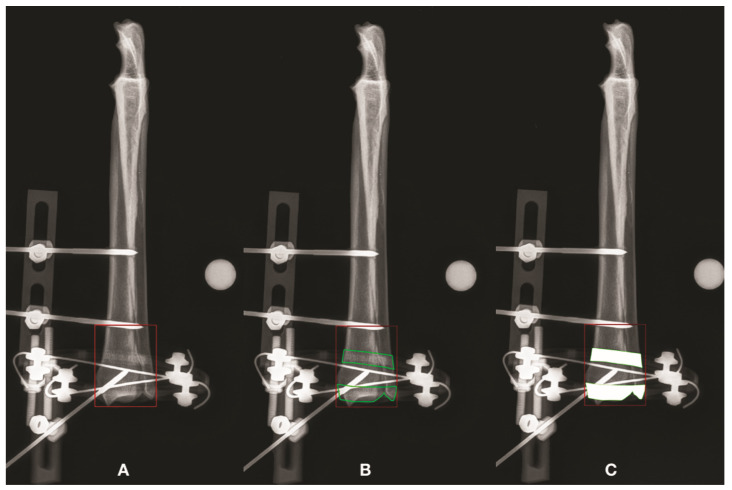
Radiographic pictures showing FA, RLA and ROA for the CrCd projection of the radius-ulna. The FA is illustrated and its extent was calculated (**A**). The RL window represents the area inside the FA where it is possible to see the bone, either not superimposed on radiopaque elements of the frame or superimposed on the radiolucent component of the frame. The latter bone areas are represented by the green areas (**B**). Finally, the radiolucent frame inside the FA was converted into solid white areas, as if it was metallic. These areas were subtracted from the FA to obtain the ROA (**C**). The same procedure was done for each bone segment and for each CrCd and ML projection.

Each construct underwent a radiographic study with two orthogonal standard views. The digital images were imported into a graphics editing program and each projection was calibrated by comparing the number of pixels of known measurement to the “mm length” of the radiopaque marker. The FA was included in a rectangle, using the same software. The rectangle that represents the FA surrounded the bone where the fracture was supposed to be and was drawn using the software tools. Two lines were orthogonally oriented to the mechanical axis of the bone, one at the most proximal (humerus and radius) or distal (tibia) part of the FA and the other tangent to the joint surface. To close the polygon, two lines were drawn parallel to the mechanical axis of the bone, tangent to the most prominent parts of the bone itself and connecting the previous lines (Figure 1). The extent of the area was then calculated using the software in both views for each bone. Inside the FA was then identified a radiolucent (RL) window and a radiopaque (RO) window. The RL represents the areas of the FA, where it was possible to see the anatomical structures of the bone not superimposed on radiopaque parts of the frame, plus those that were still visible thanks to the superimposition of the radiolucent component of the frame not considering pins or K-wires. The RL area (RLA) was then calculated by subtracting from the FA the area of any radiopaque material of the external frame, superimposed on the underlying skeletal structures. Finally, the ratio between the RLA and FA was calculated. Once the RLA was calculated, the settings of the image were altered to turn the radiolucent material in the FA into a white, metallic-like material. Using the same software, the edges of the radiolucent material in the FA were drawn, and the density inside the edges was increased to 100%, just as if the radiolucent material was metallic material. The RO area (ROA) was then calculated by the software, subtracting from the FA the area of each part of the frame superimposed on the bone in the FA that did not allow visualization of the bone not considering pins or K-wires. Finally, the ratio between the ROA and FA was calculated (Figure 3). Data were analyzed using descriptive statistics and expressed as median and range (min–max). RLA and ROA results were compared using nonparametric statistics. Specifically, the Wilcoxon test and Friedman test were used to evaluate the effect of the radiolucent material on the RLA compared to ROA. Differences were considered significant if *p* < 0.05.

## 3. Results

Eighteen frames were applied to 18 bone specimens (six humeri, six radius-ulna and six tibiae) harvested from three dogs weighing between 24 and 36 kg that had died for reasons unrelated to this study. The FA, RLA and ROA were calculated for each construct on both postoperative radiographic views.

### 3.1. Humerus

The median value of the FA in the sagittal (AP) projection was 1918.74 mm^2^ (1779.92–1969.76). The RLA had a median area of 1918.74 mm^2^ (1779.92–1969.76). The ROA had a median value of 1599.94 mm^2^ (1543.45–1838.28). The median value of the FA in the medio-lateral (ML) projection was 1379.03 mm^2^ (1227.68–1888.40). The median area in the RLA view was 893.12 mm^2^ (715.65–1870.82). The median area of the ROA view was 749.01 mm^2^ (583.74–1713.13). The measurements of the humerus are summarized in Table 1. In the AP view, the area of the RLA was 100% and the area of the ROA was 83.4% of the FA. In the ML view, the area of the RLA was 64.7% and the area of the ROA was 54.3% of the FA. A significant difference was detected between the areas of the RLA and ROA in the AP view (*p* = 0.0313) and in the ML view (*p* = 0.0313). Similarly, there was a significant difference between the areas of the RLA and ROA in the AP view (*p* = 0.0143) and in the ML view (*p* = 0.0143).

### 3.2. Radius-Ulna

The median value of the FA in the AP projection was 1785.13 mm^2^ (1587.06–2073.3). The RLA view had a median area of 1785.13 mm^2^ (1587.06–2009.08). In the ROA view, the median area was 1256.49 mm^2^ (1130.68–1435.62). The median value of the FA in the ML projection was 1204.52 mm^2^ (942.21–1431.98). The RLA view had a median area of 875.30 mm^2^ (673.30–1317.29). The median area of the ROA view was 607.92 mm^2^ (412.01–1110.59). The measurements of the radius-ulna are summarized in Table 2. In AP view the area of the RLA was 100% and the area of the ROA was 70.4% of the FA. In the ML view, the area of the RLA was 72.7% and the area of the ROA was 50.5% of the FA. Significant differences were detected between the RLA and ROA in the AP view (*p* = 0.0313) and in the ML view (*p* = 0.0313) and between the RLA and ROA in the AP view (*p* = 0.0143) and in the ML view (*p* = 0.0143).

### 3.3. Tibia

The median value of the FA in the AP projection was 2239.75 mm^2^ (1994.06–2913.71). The RLA had a median area of 2239.75 mm^2^ (1994.06–2913.71). In the ROA view the median area was 1944.49 mm^2^ (1718.91–2614.23). The median value of the FA in the ML projection was 2269.84 mm^2^ (1885.76–2553.02). The RLA area had a median value of 1878.25 mm^2^ (1448.00–2283.24). The median area of the ROA view was 1522.45 mm^2^ (1090.29–1722.48). The measurements of the tibia are summarized in Table 3. In the AP view, the area of the RLA was 100% and the area of the ROA was 86.8% of the FA. In the ML view, the area of the RLA was 82.7% and the area of the ROA 67.1% of the FA. Significant differences were detected between the RLA and ROA in the AP view (*p* = 0.0313) and in the ML view (*p* = 0.0313), and between the RLA and ROA in the AP view (*p* = 0.0143) and in the ML view (*p* = 0.0143), respectively.

## 4. Discussion

The use of external fixation has been historically associated with the interference on the radiographic visualization of the area of interest, both for fractures and osteotomies. The bone healing process has often been obscured by the fixator frame, which precludes consistent evaluation on the two orthogonal standard planes for decision-making purposes. This has been particularly true with the use of CEFs, while with LEFs, usually one projection is free of interference from the frame. The use of hybrid frames partially reduced the impact of radiographic interference, mostly due to the linear component [18,19,20]. The introduction of radiolucent materials aimed to dramatically reduce such interference, allowing bone visualization even when the fixator frame is superimposed on the area of interest [4,13,21]. The study was designed to eliminate any change between the radiolucent and metallic frame to compare that could have produced uncomparable data. The graphical simulation of the metallic frame performed on the same radiographic picture obtained with the radiolucent frame allowed to exactly compare what was radiographically visible with each construct. The frame constructs were chosen based on the most common constructs described in the literature, [16,21,22]. As usual for external fixation, the sagittal projection is less prone to heavy interference from the frame, because most of the bone-holding elements (wires and pins) lie in the medio-lateral or oblique planes, not in the cranio-caudal one. This is due to anatomical constraints to avoid impinging anatomical relevant structures. Nevertheless, the area in AP view is usually reduced in ROA visualization, due to the ring component of the hybrid frame, which is superimposed on the limb [19,20]. The medio-lateral projection is usually much more affected by interference, because this is the plane in which most of the holding elements and their related connection elements (bars, posts, rings) lie. Although the difference in results between the radiolucent and metallic frame was not more pronounced than that in the AP view in this study, the clinical consequence of this difference could be much more important. The reduction in visualization in the AP view in the ROA mode is compared to a RLA that is very often 100%, and this means that the reduction is applied to an area that is almost completely visible. The visualization in RLA mode in the ML projection is already reduced, and a further reduction in ROA mode can make visualization impossible for the area of interest. Our experimental model has some limitations. One of the most important is that intact bones were used to simulate the fracture environment, and as a result, the frame constructs were chosen based on the most common constructs described in the literature [16,21,22]. Another limitation to this study was the lack of evaluation of yield point or load-to-failure, as described in reports of mechanical studies [23,24,25,26,27]. The current study was limited to no loading in the FA, while cyclic loading is considered to be most representative of the conditions encountered during postoperative convalescence and most responsible for failure of constructs in a clinical setting [10,28,29,30]. However, evaluation of this aspect was outside the scope of the study, because the aim of this study was to compare the radiographic interference of a partially radiolucent external hybrid fixator with the same frame structure graphically changed as if it was metallic, but cyclic loading is a logical next step in assessing how these constructs will behave in a clinical setting. However, evaluation of this aspect was outside the scope of the study. A potential advantage of the use of radiolucent materials is for small and toy breed dogs, because the fracture area is usually very small, and easily obscured by the fixator components. Furthermore, radiolucent materials are usually much lighter than metals, and this can positively affect the overall weight of the final frame, helping a small patient use the limb during treatment.

## 5. Conclusions

Based on the results of this study, the use of radiolucent materials can be a valuable option for external fixation, and mostly for circular/hybrid fixation, in order to decrease the radiographic interference of the frame, allowing better assessment of fracture reduction and bone healing on postoperative radiographs. Future clinical studies to clarify the effects of this type of external fixation with radiolucent material on promoting a correct bone healing are needed, in order to be effective beyond the ability to improve the monitoring of the healing process.

## Figures and Tables

**Table 1:** Measurements of the Humerus in the antero-posterior (AP) and medio-lateral (ML) view.

Bone Segment	Case	Projection	FA (mm^2^)	RLA (mm^2^)	ROA (mm^2^)	RLA-ROA (mm^2^)	FA/RLA (%)	FA/ROA	FA/ROA (%)	RLA/ROA	RLA/ROA (%)	Statistical Result
**Humerus**												
	1	AP	1958.33	1958.33	1838.28	120.06	1.00 (100)	1.07	107	1.07	107	
	1	ML	1888.4	1870.82	1713.13	157.69	1.01 (101)	1.1	110	1.09	109	
	2	AP	1966.67	1966.67	1581.47	385.2	1.00 (100)	1.24	124	1.24	124	
	2	ML	1404.31	931.4	752.07	179.33	1.51 (151)	1.87	187	1.24	124	
	3	AP	1969.76	1969.76	1543.45	426.3	1.00 (100)	1.28	128	1.28	128	
	3	ML	1227.68	715.65	583.74	131.92	1.72 (172)	2.1	210	1.23	123	
	4	AP	1824.27	1824.27	1627.47	196.8	1.00 (100)	1.12	112	1.12	112	
	4	ML	1421.43	854.83	745.95	108.88	1.66 (166)	1.91	191	1.15	115	
	5	AP	1879.15	1879.15	1618.41	260.74	1.00 (100)	1.16	116	1.16	116	
	5	ML	1353.75	1138.71	1053.82	84.89	1.19 (119)	1.28	128	1.08	108	
	6	AP	1779.92	1779.92	1553.29	226.63	1.00 (100)	1.15	115	1.15	115	
	6	ML	1315.31	839.31	724.42	114.89	1.57 (157)	1.82	182	1.16	116	
**Median**		AP	1918.74	1918.74	1599.94	243.68	1	1.15	115.35	1.15	115.35	*p* = 0.0143
		ML	1379.03	893.12	749.01	123.4	1.54	1.84	184.15	1.15	115.23	*p* = 0.0143
**(range)**		AP	(1779.92–1969.76)	(1779.92–1969.76)	(1543.45–1838.28)	(120.06–426.30)	(1.00–1,00)	(1.07–1.28)	(106.53–127.62)	(1.07–1.28)	(106.53–127.62)	
		ML	(1227.68–1888.40)	(715.65–1870.82)	(583.74–17,134.13)	(84.89–179.33)	(1.01–1.72)	(1.10–2.10)	(110.23–210.31)	(1.08–1.24)	(108.06–123.84)	

Antero-posterior (AP), medio-lateral (ML), fracture area (FA), radiolucent area (RLA), radiopaque area (ROA), square millimeters (mm^2^), percentage (%).

**Table 2:** Measurements of the radius-ulna in the antero-posterior (AP) and medio-lateral (ML) view.

Bone Segment	Case	Projection	FA (mm^2^)	RLA (mm^2^)	ROA (mm^2^)	RLA-ROA (mm^2^)	FA/RLA (%)	FA/ROA	FA/ROA (%)	RLA/ROA	RLA/ROA (%)	Statistical Result
**Radius-Ulna**												
	1	AP	2009.08	2009.08	1435.62	573.46	1.00 (100)	1.4	140	1.4	140	
	1	ML	1221.11	949.46	731.71	217.75	1.29 (129)	1.67	167	1.3	130	
	2	AP	2073.31	1962.72	1384.6	578.12	1.06 (106)	1.5	150	1.42	142	
	2	ML	1333.83	1317.29	1110.59	206.69	1.01 (101)	1.2	120	1.19	119	
	3	AP	1587.06	1587.06	1130.68	456.39	1.00 (100)	1.4	140	1.4	140	
	3	ML	1020.26	745.04	440.14	304.9	1.37 (137)	2.32	232	1.69	169	
	4	AP	1823.71	1823.71	1204.22	619.49	1.00 (100)	1.51	151	1.51	151	
	4	ML	1187.93	801.14	484.13	317.01	1.48 (148)	2.45	245	1.65	165	
	5	AP	1661.69	1661.69	1232.98	428.71	1.00 (100)	1.35	135	1.35	135	
	5	ML	942.22	673.3	412.01	261.29	1.40 (140)	2.29	229	1.63	163	
	6	AP	2009.08	2009.08	1435.62	573.46	1.00 (100)	1.4	140	1.4	140	
	6	ML	1431.98	1295.45	761.4	534.05	1.11 (111)	1.88	188	1.7	170	
**Median**		AP	1785.13	1785.13	1256.49	520.01	1	1.4	140.15	1.4	140.15	*p* = 0.0143
		ML	1204.52	857.3	607.92	283.1	1.33	2.08	208.38	1.64	164.45	*p* = 0.0143
**(range)**		AP	(1587.06–2073.31)	(1587.06–2009.08)	(1130.68–1435.62)	(428.71–619.49)	(1.00–1.06)	(1.35–1.51)	(134.77–151.44)	(1.35–1.51)	(134.77–151.44)	
		ML	(942.22–1431.98)	(673.30–1317.29)	(412.01–1110.59)	(206.69–534.05)	(1.01–1.48)	(1.20–2.45)	(120.10–245.38)	(1.19–1.70)	(118.61–170.14)	

Antero-posterior (AP), medio-lateral (ML) fracture area (FA), radiolucent area (RLA), radiopaque area (ROA), square millimeters (mm^2^), percentage (%).

**Table 3:** Measurements of the tibia in the antero-posterior and medio-lateral view.

Bone Segment	Case	Projection	FA (mm^2^)	RLA (mm^2^)	ROA (mm^2^)	RLA-ROA (mm^2^)	FA/RLA (%)	FA/ROA	FA/ROA (%)	RLA/ROA	RLA/ROA (%)	Statistical Result
**Tibia**												
	1	AP	2448.26	2448.26	2193.1	255.16	1.00 (100)	1.12	112	1.12	112	
	1	ML	2368.11	1858.46	1594.34	264.12	1.27 (127)	1.49	149	1.17	117	
	2	AP	2181.06	2181.06	1906.42	274.64	1.00 (100)	1.14	114	1.14	114	
	2	ML	2522.34	2118.95	1532.54	586.41	1.19 (119)	1.65	165	1.38	138	
	3	AP	2034.36	2034.36	1771.65	262.71	1.00 (100)	1.15	115	1.15	115	
	3	ML	1885.76	1448	1090.29	357.72	1.30 (130)	1.73	173	1.33	133	
	4	AP	1994.06	1994.06	1718.91	275.15	1.00 (100)	1.16	116	1.16	116	
	4	ML	1917.27	1636.64	1241.88	394.76	1.17 (117)	1.54	154	1.32	132	
	5	AP	2298.43	2298.43	1982.56	315.87	1.00 (100)	1.16	116	1.16	116	
	5	ML	2171.56	1898.04	1512.37	385.67	1.14 (114)	1.44	144	1.26	126	
	6	AP	2913.71	2913.71	2614.23	299.48	1.00 (100)	1.11	111	1.11	111	
	6	ML	2553.02	2283.24	1722.48	560.76	1.12 (112)	1.48	148	1.33	133	
**Median**		AP	2239.75	2239.75	1944.49	274.89	1	1.15	114.62	1.15	114.62	*p* = 0.0143
		ML	2269.84	1878.25	1522.45	390.21	1.18	1.51	151.46	1.32	132.17	*p* = 0.0143
**(range)**		AP	(1994.06–2913.71)	(1994.06–2913.71)	(1718.91–2614.23)	(255.16–315.87)	(1.00–1.00)	(1.11–1.16)	(111.46–116.01)	(1.11–1.16)	(111.46–116.01)	
		ML	(1885.76–2553.02)	(1448.00–2283.24)	(1090.29–1722.48)	(264.12–586.41)	(1.12–1.30)	(1.44–1.73)	(143.59–172.96)	(1.17–1.38)	(116.57–138.26)	

Antero-posterior (AP), medio-lateral (ML), fracture area (FA), radiolucent area (RLA), radiopaque area (ROA), square millimeters (mm^2^), percentage (%).

## Data Availability

The data presented in the study are available in the manuscript.
